# Recovery and Preparation of Innovative Products and Composite Materials for Environmental Applications

**DOI:** 10.3390/ma18245681

**Published:** 2025-12-18

**Authors:** Agnieszka Generowicz, Krzysztof Barbusiński, Maciej Thomas

**Affiliations:** 1 Cracow University of Technology, Faculty of Environmental Engineering and Energy, Warszawska 24, 31-155 Cracow, Poland; maciej.thomas@pk.edu.pl; 2 Silesian University of Technology, Department of Water and Wastewater Engineering, Konarskiego 18, 44-100 Gliwice, Poland

## 1. Introduction

The articles published in this Special Issue of the journal *Materials*, titled “Recovery and Preparation of Innovative Products and Composite Materials for Environmental Applications,” discuss the recovery and recycling of materials in the environmental field. They encompass two main areas of research:

- The production of low-cost adsorbents from waste biomass;
- The recovery of rare-earth elements from sewage sludge and hazardous waste.

Waste and sludge management is one of the main elements of a circular city, and it involves the management of material and energy cycles [1,2]. Closing cycles in a city, agglomeration, or region allows for the creation and closure of metabolic cycles by introducing a coordinated structure containing interconnected streams of mass, energy, information, and functional technical elements (e.g., technologies) that cooperate in the implementation of a circular economy [3]. Decision-making regarding the following areas requires detailed and extensive analyses, as well as advanced tools, that consider many variable factors and parameters [4,5,6,7]: selection of the forms of waste to recover, recycle, or dispose of via various methods; the design of processing installations; assessments of the efficiency and so-called ecological effects of installations or entire systems; financial arrangements; the balancing of material or energy flows from processing procedures; and the forecasting of changes in the nature of the waste or the quality of the products manufactured from it.

The increasing environmental demands that are being placed and the limited amount of raw material resources at our disposal now, as well as the presence of multiple environmental crises, i.e., climate and energy crises, are encouraging a search for technologies that will help us achieve reductions in waste while simultaneously recovering and recycling raw materials [1,6,7]. Research on the various types and streams of waste from various technologies, as well as their use as raw materials for new or existing technologies, investigations into the use of byproducts, and studies exploring the status of waste loss to identify new products, raw materials or materials are examples of the “waste-to-resource” approach, which is consistent with the concept of the circular economy [8,9,10].

Implementing a circular economy requires assessments of its cost-effectiveness, environmental impact, and social acceptance, all of which are integrated into sustainable development assumptions, thus embracing the creation of a resource-efficient, energy-secure and socially secure economy. Such solutions require experimental analyses and interdisciplinary evaluations at the laboratory scale, semitechnical scale, and large-scale industrial levels. Faced with increasing pressures resulting from raw material consumption, waste generation, and pollution, recovery and recycling are becoming key technological factors enabling the extraction of valuable raw material fractions. This poses new challenges for waste management, as well as for recovery and recycling processes. With increasing consumerism, manufacturers tend to constantly encourage the purchase of new products, and the production of increasingly less durable goods accelerates the consumption of natural resources while simultaneously requiring the processing of existing ones. When unmanaged secondary raw materials migrate into the environment, they generate emissions and exacerbate already existing negative environmental impacts while also causing economic losses.

Moreover, reusing secondary raw materials and waste reduces the amount of resources extracted, reduces the emissions associated with production and mining, and limits the amount of waste ending up in landfills or in the environment. Therefore, recycling should be considered a waste management method aimed at generating new value streams that can be used in new environmental applications [11,12,13]. Numerous technological and scientific studies [8,9,13,14,15] are continuing to present new solutions, not only demonstrating the potential for their implementation but also introducing techniques for environmental, economic, and administrative assessments [16,17].

## 2. Research Areas for New Technologies and Products Focused on Environmental Solutions

### 2.1. Low-Cost Adsorbents from Waste Biomass—Examples and Potential

In a proposed solution [18], biomass adsorbents were developed that were synthesized from sodium alginate mixed with various banana peel-based materials, activated carbon, and magnetic activated carbon. These adsorbents were evaluated for their effectiveness in removing tetracycline and hexavalent chromium as model contaminants, representing antibiotics and heavy metals, respectively. Characterization of the adsorbents revealed that functional groups and thermal stability favour effective adsorption, whereas adsorption tests revealed removal efficiencies of up to 92% for tetracyclines and 79% for chromium. The adsorbents retained significant adsorption capacity in subsequent reuse cycles, indicating their regenerative potential.

In [19], the adsorption efficiency of activated carbon was compared with that of three fractions (greater than 1.6 mm, greater than 0.16 mm, and less than 0.16 mm) of a biosorbent made from crushed Adansonia digitata shells.

The adsorbents were prepared and characterized via TGA, SEM, EDX, FTIR, and pH-PZC analyses. Drug adsorption tests (phenobarbital) of aqueous solutions have shown that biosorbents/activated carbon from plant shells can be relatively effective in removing such contaminants.

In a subsequent study [20], the authors investigated the possibility of producing an activated carbon substitute on the basis of pretreated lignocellulosic biomass, particularly spruce sawdust. Harmful liquid waste from desalination was used to process solid waste from the wood industry, spruce sawdust. The processed sawdust was tested as an adsorbent for removing methylene blue from industrial wastewater. The adsorption capacity of the pretreated material was fourfold greater than that of the untreated material. The brine modification process changed the porous structure and chemical properties of the sawdust, increasing the number of active adsorption sites. Furthermore, a kinetic experiment was conducted on the methylene blue adsorption process. The adsorption capacity of sawdust pretreated with desalinated brine was approximately twofold greater than that of untreated sawdust, as determined via a second-order kinetic equation that best fit the kinetic data of the three kinetic models employed in this study. An industrial-scale solution was also proposed on the basis of kinetic data, including optimal conditions for the pretreatment of spruce sawdust under experimentally determined conditions.

The studies described in [18,19,20] concerning the use of organic wastes from agri-food sources show that, after appropriate treatment (chemical modification, activation, and shaping), they can be effective sorbents for environmental pollutants such as dyes and drugs. Importantly, experimental studies have demonstrated that these materials not only have good sorption capacity but also have the advantages of low raw material costs and positive environmental profiles compared with conventional sorbents, as well as the possibility of sorbent regeneration/recovery. The demonstrated strengths of these solutions perfectly align these studies with the philosophy of the circular economy.

### 2.2. Recovery of Selected Rare Earth Metals from Sewage Sludge Resulting from Brewery Wastewater Treatment or from Waste from Used Lithium-Ion Batteries

Owing to the high costs and limited sources of cerium coagulants, recycling processes are becoming increasingly important. Cerium is a rare-earth metal used in exhaust gas catalytic converters, electronics, and optical materials, among other applications. The use of cerium coagulant facilitates wastewater treatment, but it creates sludge rich in cerium, which can be lost and released into the environment without recovery. Studies have shown that it is possible to recover cerium in the form of soluble salts, closing the material cycle in wastewater treatment, reducing the amount of sewage sludge, and reusing recovered cerium as a coagulant. The research in [21] presented a new technology for the recovery of cerium(III) chloride, cerium(III) sulfate, and cerium(IV) sulfate and, potentially, phosphate from sewage sludge (101.5 g/kg Ce and 22.2 g/kg total P) in the treatment of brewery wastewater using recovered CeCl_3_ as a coagulant. The study was preceded by an analysis of the chemical composition of brewery sewage sludge obtained via coagulation using recycled CeCl_3_, which demonstrated that, owing to the high concentrations of Ce and P (101.5 g/kg Ce and 22.2 g/kg P), the waste can be used as a raw material in the recovery of these elements. Under optimal conditions (0.35 g HCl per 1 g of sludge, a reaction time of 40 min, and an extractant volume of 25 mL per 1 g of sludge), the highest recycling levels achieved were 99.6% and 97.5% for Ce and P, respectively. The filtrate obtained after filtering out Ce_2_(C_2_O_4_)_3_·10H_2_O contained 570 mg/L phosphorus, which enabled its use as a source of phosphorus compounds.

The proposed sewage sludge processing method represents an innovative way to reuse waste, where individual processes generate a range of products that could be reused in areas such as municipal or industrial waste disposal, the remediation of degraded industrial sites, transport (e.g., road deicing), and even agriculture. Importantly, from a technological perspective, these processes must be designed to minimize metal losses in subsequent cycles (such as low lithium losses in hydrometallurgical processes), and the economic viability of metal recovery depends on the metal concentration in the waste stream.

In [22], components of the pyrohydrometallurgical processing of spent lithium-ion batteries were investigated, including the hydrometallurgical treatment of lithium slag and the refining of the resulting leachate. Leaching was performed by dry fermentation, which is an effective method that enables the transfer of more than 99% of the metals present, such as Li, Al, Co, and Cu, to the leachate. In this work, the effects of three types of precipitating agents (NaOH, NH_4_OH, and Na_3_PO_4_) on the precipitation efficiency of Al and Li losses were investigated. It was found that aluminum precipitation with NaOH can lead to lithium coprecipitation, resulting in a total lithium loss of up to 40%. Crystalline Na_3_PO_4_ was selected as a suitable precipitating agent for the complete removal of Al from the Li leachate with minimal lithium loss (less than 2%) under the following conditions: pH 3, 400 rpm, 10 min, and room temperature. Analysis confirmed that, in addition to aluminium, the sediment also contained the rare-earth elements (REEs) La (3.4%), Ce (2.5%), Y (1.3%), Nd (1%), and Pr (0.3%). The selective recovery of these elements should be the subject of further studies.

The recycling of rare-earth metals is becoming increasingly crucial because of the high cost of their extraction and the increasingly limited availability of these resources. The research in [21,22] emphasized the importance of designing recovery technologies for minimizing metal losses and increasing the profitability of recycling processes.

## 3. Implementation Barriers and Challenges for Recovery, Recycling and New Material Applications

The implementation of recovery and recycling technologies is becoming a significant economic and social challenge, primarily because of costs and technological constraints. These constraints and challenges can be grouped into several categories:

- Technological risks, including the immaturity and imperfection of technologies that are still at the laboratory or pilot stage, material losses in subsequent stages of recovery and recycling processes, the lack of infrastructure at the technological level, and the low concentration of raw materials in waste.
- Economic risks, including high investment costs, uncertain financial returns and profitability, depend mainly on the market prices of raw materials and secondary raw materials, which are subject to high volatility, competition with cheaper primary raw materials, especially if they come from countries with lower environmental standards, and the high energy consumption and operating costs of recycling installations, which are often more expensive and energy intensive than primary production [23].
- Regulatory and administrative risks, such as the variability in and diversity of legal regulations in individual countries, the lack of uniform quality standards for recovered raw materials and new products, which makes it difficult to reintroduce them to the market, extensive bureaucracy, which causes difficulties in obtaining environmental permits, and the risk of “greenwashing”, in which companies may declare the circularity of activities that, in fact, do not reduce, or may even increase, the burden on the environment.
- Environmental risks, which may include the improper management of waste from recycling processes; the formation of unsuitable or toxic fractions; the risk of secondary emissions—where some recovery processes may result in pollutant emissions that require additional treatment—the consumption of energy, water and other natural resources; and the lack of a uniform methodology for calculating the environmental impact of new technologies, products, materials and substances [23], for example.
- Social risks, such as low levels of social acceptance resulting from a lack of knowledge, as well as a lack of competences, because transformation requires trained interdisciplinary staff with new qualifications and changes in consumption models, which require education and changes in social habits.
- Risks related to supply chains include the low quality of raw materials from waste, the variable composition of waste streams, which hinders stable production, a lack of cooperation between economic sectors, and unpredictable waste supply.

## 4. Conclusions

The intensive development of the industry in recent years has resulted in an increase in the demand for raw materials to produce goods for the functioning of modern civilizations. This requires the use of increasingly advanced production technologies, ensuring the optimal use of raw materials, water, electricity, and human labor. However, industrial development also causes negative changes in the environment. For this reason, environmental issues remain a significant problem requiring comprehensive analysis and solutions.

Despite the relatively small number of articles in this special issue, these publications cover the very important issues of material recovery and recycling in the areas of environmental engineering and the circular economy.

The presented examples and research findings demonstrate that the recovery and recycling of industrial waste or sewage sludge are key elements of sustainable development strategies and provide practical examples of the circular economy concept. These activities transform waste into a raw material, helping reduce the exploitation of primary resources and the associated negative environmental impacts, provided that the infrastructure is cost-effective and expands to ensure that industrial-scale products are reproducible and durable.
